# Supplementary material for: Evaluation of the test accuracy of a SARS-CoV-2 rapid antigen test in symptomatic community dwelling individuals in the Netherlands
Source: PLoS One. 2021 May 13;16(5):e0250886. doi: 10.1371/journal.pone.0250886 (PMC8118553; doi:10.1371/journal.pone.0250886)
Supplement: Attachment — Submitted filename: Response to Reviewers.docx [file pone.0250886.s003.docx]

Dear Editor,

We would like to thank you for the second evaluation of our manuscript 'Performance evaluation of a SARS-CoV-2 rapid antigen test: test performance in the community in the Netherlands' (PONE-D-20-36174) and the opportunity to resubmit a revised copy. We are grateful for the additional corrections and comments.

Please find the responses to the points raised - following the original comment in italics - beneath. Two versions of the revised manuscript, one with and one without track changes, were uploaded alongside with this document.

Sincerely yours,

Nathalie Van der Moeren
on behalf of the co-authors

Additional points raised by Reviewer 2

-Line 184 tracked changes version: (full participant information letter on the website, ...): will there be an url address be added?

*Thank you for this comment. As the participant information letter was written in Dutch, we doubt the added value of adding the URL. (https://www.google.com/url?sa=t&rct=j&q=&esrc=s&source=web&cd=&ved=2ahUKEwjnt9aWydrvAhUIg_0HHc0aAaUQFjAAegQIAxAD&url=https%3A%2F%2Fwww.ggdwestbrabant.nl%2Fcoronavirus%2F-%2Fmedia%2FA70F8404BCC24075B03934AA764D9FBD.ashx&usg=AOvVaw1Hx9cTDPy_qh-0miug_rY0)*

-line 186: "No written informed consent was obtained as this would have compromised the strictly needed high flow of individuals being tested at...": the explanation is not necessary here - as long as this was discussed with the ethical committee and the written consent was waved, then this is valid. It is written later in the text as well. NOt necessary here.

*Thank you for this comment. The extra information on why no written informed consent was obtained was added to the manuscript at the request of reviewer 2 and the academic editor and was clarified for study part one and two seperately.*

-line 233: "he cut-off of 7 days was chosen based...": results OF a study by Bullard... (some re-writing - English- of the sentence necessary)

*Thank you for this remark, the sentence was rewritten (line 206).*

-line 288: "18 to 83 years (M = 44,": mean or median?

*M stands for mean, this was added to the manuscript (line259).*

-line 296: "to be the ": take out the (and as well from line 301)

*Thank you for this correction, ‘the’ was deleted from both lines. (line 263 and 267)*

-line 337: "Based the cohort in part two of the study":re-write - word missing...

*Thank you for this correction, the sentence was adapted. (line 286)*

-line 384: "overestimate test accuracy in final clinical setting.".. grammar

*The sentence was rewritten. (line 324)*
