# Supplementary material for: Evaluation of the test accuracy of a SARS-CoV-2 rapid antigen test in symptomatic community dwelling individuals in the Netherlands
Source: PLoS One. 2021 May 13;16(5):e0250886. doi: 10.1371/journal.pone.0250886 (PMC8118553; doi:10.1371/journal.pone.0250886)
Supplement: Attachment — Submitted filename: Response to Reviewers.docx [file pone.0250886.s004.docx]

Dear Editor,

We would like to thank you for the chance to resubmit a third revised copy of our manuscript 'Performance evaluation of a SARS-CoV-2 rapid antigen test: test performance in the community in the Netherlands' (PONE-D-20-36174). We are grateful for the additional corrections and comments.

Please find the responses to the points raised - following the original comment in italics - beneath. Two versions of the revised manuscript, one with and one without track changes, were uploaded alongside with this document.

Sincerely yours,

Nathalie Van der Moeren
on behalf of the co-authors

Additional points raised by the Editor

- PLOS ONE does not copy edit accepted manuscripts. We therefore request a minor modification of the statement in the ethics section lines 158-159" In part one of the study, individuals were informed about the study **trough** local media, by 159 MHS communication channels (full participant information letter on the **website, …)**. We accept not adding the URL as the letter will be in Dutch. However, the statement as it is, (website.....) may confuse readers into thinking there is missing data. Kindly delete the dots after website.

*The dots in question were removed. (line 159)*

- In addition please change the word trough to through.

  *Thank you for this correction, trough was changed by through. (line 158)*
- A final spelling and grammar check for your manuscript will be appreciated before you resubmit.

*We performed a final spelling and grammar check, minor corrections were made.*
